# (2*SR*,3*RS*)-Methyl 2-(adamantan-1-yl)-3-phenyl­sulfonyl-3-(pyridin-2-ylsulfan­yl)propano­ate dichloro­methane hemisolvate

**DOI:** 10.1107/S1600536811010312

**Published:** 2011-03-26

**Authors:** Rosa-Luisa Meza-León, Sylvain Bernès, Elsie Ramírez Domínguez, Martha Sosa-Rivadeneyra, Leticia Quintero-Cortés

**Affiliations:** a Centro de Investigación de la Facultad de Ciencias Químicas, Universidad Autónoma de Puebla, 72570 Puebla, Pue., Mexico; b DEP Facultad de Ciencias Químicas, UANL, Guerrero y Progreso S/N, Col. Treviño, 64570 Monterrey, NL, Mexico

## Abstract

The title compound, C_25_H_29_NO_4_S_2_ 0.5CH_2_Cl_2_, was obtained as a racemate. The pyridine and phenyl rings are arranged face-to-face, giving a weak intra­molecular π–π inter­action [centroid–centroid separation = 3.759 (3) Å]. These inter­actions are extended inter­molecularly, forming chains of stacked rings along [001] with separations of 3.859 (3) and 3.916 (3) Å. The solvent used for crystallization, CH_2_Cl_2_, is located in voids between the chains of mol­ecules, with a site occupancy of 0.5.

## Related literature

For chemical, polymer and pharmaceutical applications of adamantane and its derivatives, see: Beller *et al.* (2002 ▶); Mathias *et al.* (1995 ▶, 2001 ▶); Stotskaya *et al.* (1995 ▶); Spasov *et al.* (2000 ▶); Enomoto *et al.* (2010 ▶). For catalyst reactions, see: Taoufik *et al.* (1999 ▶). For poly(*p*-phenyl­ene­vinyl­ene) (PPV) derivatives, see: Jeong *et al.* (2002 ▶). For their anti­viral and disease-related activity, see: Kadi *et al.* (2010 ▶); Papanastasiou *et al.* (2010 ▶) and for their use in the treatment of influenza A, leukemia and deafness, see: Zarubaev *et al.* (2010 ▶); Spasov *et al.* (2000 ▶). For the Barton deca­rboxylation reaction, see: Togo (2004 ▶).
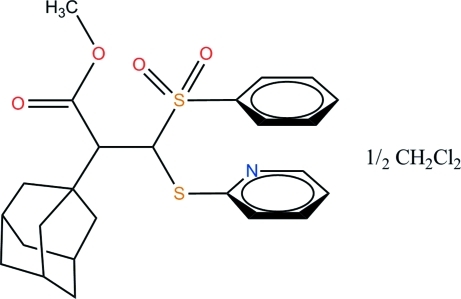


## Experimental

### 

#### Crystal data

C_25_H_29_NO_4_S_2_·0.5CH_2_Cl_2_
*M* *_r_* = 514.08
Monoclinic, 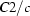

*a* = 12.709 (4) Å
*b* = 27.820 (6) Å
*c* = 14.448 (3) Å
β = 101.254 (19)°
*V* = 5010 (2) Å^3^
*Z* = 8
Mo *K*α radiation
μ = 0.35 mm^−1^
*T* = 298 K
0.40 × 0.40 × 0.40 mm

#### Data collection

Siemens P4 diffractometer
6603 measured reflections
4434 independent reflections
2936 reflections with *I* > 2σ(*I*)
*R*_int_ = 0.026
3 standard reflections every 97 reflections  intensity decay: 40%

#### Refinement

*R*[*F* ^2^ > 2σ(*F* ^2^)] = 0.054
*wR*(*F* ^2^) = 0.154
*S* = 1.03
4434 reflections
316 parameters
H-atom parameters constrained
Δρ_max_ = 0.41 e Å^−3^
Δρ_min_ = −0.31 e Å^−3^

### 

Data collection: *XSCANS* (Siemens, 1996 ▶); cell refinement: *XSCANS*; data reduction: *XSCANS*; program(s) used to solve structure: *SHELXTL* (Sheldrick, 2008 ▶); program(s) used to refine structure: *SHELXTL*; molecular graphics: *SHELXTL* and *Mercury* (Macrae *et al.*, 2008 ▶); software used to prepare material for publication: *SHELXTL*.

## Supplementary Material

Crystal structure: contains datablocks I, global. DOI: 10.1107/S1600536811010312/jj2070sup1.cif

Structure factors: contains datablocks I. DOI: 10.1107/S1600536811010312/jj2070Isup2.hkl

Additional supplementary materials:  crystallographic information; 3D view; checkCIF report

## Figures and Tables

**Fig. 1.**
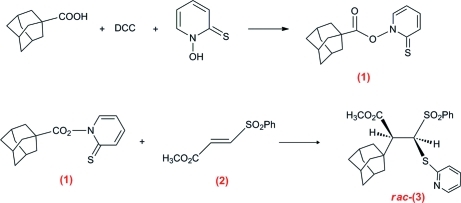
Synthetic route for the title compound.

**Fig. 2.**
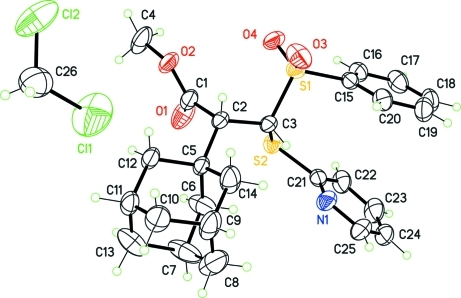
The structure of the title compound, with displacement ellipsoids at the 30% probability level for non-H atoms.

**Fig. 3.**
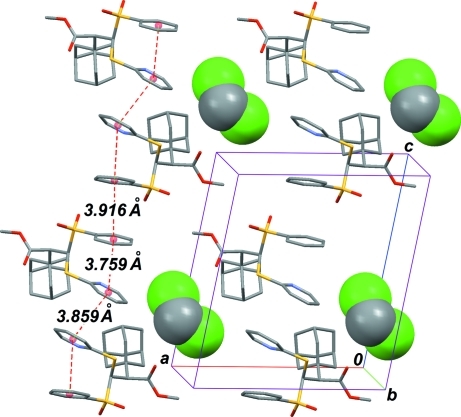
Packing diagram of (I) viewed down the b axis. Distances for intra- and inter-molecular π–π interactions are labeled for one stack of molecules along the c axis. The solvent molecules are shown as spacefilled and the H atoms are omitted for clarity.

## supplementary crystallographic information

### Comment

Adamantane and its derivatives have a broad range of chemical (Taoufik *et al.*, 1999; Beller *et al.*, 2002), polymer (Mathias *et al.*, 1995, 2001), and pharmaceutical (Stotskaya *et al.*, 1995; Spasov *et al.*, 2000; Enomoto *et al.*, 2010) applications. Compounds containing adamantyl radicals are useful catalysts for many chemical reactions, such as the refining of halogen atoms and preparation of heterogeneous bimetallic catalysts (Taoufik *et al.*, 1999). The rigid, spherical shape of adamantane reduces interchain interactions in polymers and may help with the synthesis of poly(*p*-phenylenevinylene) (PPV) derivatives (Jeong *et al.*, 2002). Adamantane-containing molecules have also been found to have antiviral activity (Kadi *et al.*, 2010; Papanastasiou *et al.*, 2010) and have been used in the treatment of influenza A (Zarubaev *et al.*, 2010), HIV-1, leukemia and deafness (Spasov *et al.*, 2000).

Alkyl radicals derived from *O*-acyl esters of *N*-hydroxy-2-thiopyridone (*a.k.a*. Barton esters) are nucleophilic, so treatment with electron-deficient olefins such as vinyl sulfones generates the corresponding addition products (alkyl 2-pyridyl sulfides) effectively.  Derivatives generated from adamantylcarboxylic acid using the Barton method (Togo, 2004) have potential biological activity. Crystallization of the racemate in the title compound is similar to an *anti* addition of the Barton ester to the olefin.

The title compound, is a racemic mixture of enantiomers (Fig. 2). The CH_2_Cl_2_ solvent molecule is placed close to a 2-fold axis with a site occupancy of 1/2.  The dihedral angle between the mean planes of the phenyl and pyridine rings is 20.24 (12)° [centroid to centroid separation = 3.759 (3)Å].  This π–π intramolecular interaction is extended along the *c* axis, with intermolecular pyridine-pyridine and phenyl-phenyl interactions related by 2-fold symmetry.  Distances separating rings are 3.859 (3)Å and 3.916 (3)Å, respectively, while angles between aromatic mean planes are 25.28 (13)° and 19.84 (7)° (Fig. 3).  CH_2_Cl_2_ molecules are placed between the chains of molecules stacked through these π–π contacts.

### Experimental

To a solution of 1,3-dicyclohexylcarbodiimide (DCC, 2 mmol) in CH_2_Cl_2_ (8 ml) was added *N*-hydroxy-2-thiopyridone (2.2 mmol) under an argon atmosphere. The solution was protected from light with aluminium foil and kept at 273 K in an ice bath. Adamantylcarboxylic acid (2 mmol) dissolved in CH_2_Cl_2_ was added dropwise to the solution. After the addition, the mixture was allowed to reach room temperature and further stirred for a period of 1.5 h. The resulting yellow solid was filtered on a bed of silica gel and washed with dry CH_2_Cl_2_ (all in dark). The filtrate was concentrated under reduced pressure, to give a crystalline solid. m.p. 164–166°C (compound 1 in Fig. 1). *O*-acyl ester 1 (1 mmol) was dissolved in CH_2_Cl_2_ (5 ml) under an argon atmosphere and (*E*)-methyl-3-(phenylsulfonyl)acrylate 2 (1.1 mmol) was added to the yellowish solution. The mixture was irradiated with a tungsten lamp (150 W), following the reaction by TLC. The products were purified by chromatography on silica gel (eluent: hexane:ethyl-acetate, 7:3). A white crystalline solid was obtained with a yield of 88%. m.p. 145–146 °C (compound 3).This compound was crystallized by slow evaporation of a CH_2_Cl_2_ solution, affording the title hemisolvate.

### Refinement

Crystals of the title hemisolvate are stable in air for months, but solvent loss occurs under X-ray irradiation. A complete data set for the studied crystal was however collected over a period of 54 h, during which the intensity decayed by *ca*. 40%. Raw data were corrected using three periodically measured reflections. All H atoms were placed in idealized positions, with C—H bond lengths fixed to 0.93 (aromatic CH), 0.96 (methyl CH_3_), 0.97 (methylene CH_2_), or 0.98 Å (methine CH). Isotropic displacement parameters for H atoms were computed as *U*_iso_(H) = 1.5 *U*_eq_(carrier C) for the methyl group and *U*_iso_(H) = 1.2 *U*_eq_(carrier C) for other H atoms.

### Crystal data

**Table:**

C_25_H_29_NO_4_S_2_·0.5CH_2_Cl_2_	*F*(000) = 2168
*M**_r_* = 514.08	*D*_x_ = 1.363 Mg m^−^^3^
Monoclinic, *C*2/*c*	Melting point: 418 K
Hall symbol: -C 2yc	Mo *K*α radiation, λ = 0.71073 Å
*a* = 12.709 (4) Å	Cell parameters from 78 reflections
*b* = 27.820 (6) Å	θ = 4.8–12.4°
*c* = 14.448 (3) Å	µ = 0.35 mm^−^^1^
β = 101.254 (19)°	*T* = 298 K
*V* = 5010 (2) Å^3^	Irregular, colourless
*Z* = 8	0.40 × 0.40 × 0.40 mm

### Data collection

**Table:**

Siemens P4 diffractometer	*R*_int_ = 0.026
Radiation source: fine-focus sealed tube, FN4	θ_max_ = 25.1°, θ_min_ = 1.8°
graphite	*h* = −15→4
2θ/ω scans	*k* = −33→33
6603 measured reflections	*l* = −17→17
4434 independent reflections	3 standard reflections every 97 reflections
2936 reflections with *I* > 2σ(*I*)	intensity decay: 40%

### Refinement

**Table:**

Refinement on *F*^2^	Primary atom site location: structure-invariant direct methods
Least-squares matrix: full	Secondary atom site location: difference Fourier map
*R*[*F*^2^ > 2σ(*F*^2^)] = 0.054	Hydrogen site location: inferred from neighbouring sites
*wR*(*F*^2^) = 0.154	H-atom parameters constrained
*S* = 1.03	*w* = 1/[σ^2^(*F*_o_^2^) + (0.0607*P*)^2^ + 7.2667*P*] where *P* = (*F*_o_^2^ + 2*F*_c_^2^)/3
4434 reflections	(Δ/σ)_max_ < 0.001
316 parameters	Δρ_max_ = 0.41 e Å^−^^3^
0 restraints	Δρ_min_ = −0.31 e Å^−^^3^
0 constraints	

### Fractional atomic coordinates and isotropic or equivalent isotropic displacement parameters (Å^2^)

**Table:**

	*x*	*y*	*z*	*U*_iso_*/*U*_eq_	Occ. (<1)
S1	0.17793 (7)	0.34186 (3)	0.15045 (6)	0.0583 (3)	
S2	0.17440 (7)	0.32202 (3)	−0.05013 (6)	0.0564 (3)	
N1	−0.0193 (2)	0.36197 (12)	−0.1021 (2)	0.0697 (8)	
O1	0.3954 (2)	0.35456 (12)	−0.0133 (3)	0.1005 (11)	
O2	0.44844 (18)	0.38921 (10)	0.1263 (2)	0.0777 (8)	
O3	0.1643 (3)	0.38017 (10)	0.21275 (18)	0.0860 (9)	
O4	0.2695 (2)	0.31153 (10)	0.1755 (2)	0.0830 (8)	
C1	0.3772 (3)	0.37941 (13)	0.0485 (3)	0.0607 (9)	
C2	0.2724 (2)	0.40456 (11)	0.0484 (2)	0.0475 (7)	
H2A	0.2785	0.4182	0.1117	0.057*	
C3	0.1791 (2)	0.36853 (11)	0.0372 (2)	0.0465 (7)	
H3A	0.1127	0.3871	0.0198	0.056*	
C4	0.5495 (3)	0.36437 (17)	0.1363 (4)	0.1094 (19)	
H4A	0.5953	0.3738	0.1944	0.164*	
H4B	0.5831	0.3724	0.0843	0.164*	
H4C	0.5374	0.3303	0.1368	0.164*	
C5	0.2526 (2)	0.44789 (11)	−0.0205 (2)	0.0490 (8)	
C6	0.2242 (5)	0.43425 (15)	−0.1234 (3)	0.0962 (15)	
H6A	0.1620	0.4133	−0.1340	0.115*	
H6B	0.2835	0.4169	−0.1411	0.115*	
C7	0.1992 (6)	0.48093 (18)	−0.1860 (3)	0.125 (2)	
H7A	0.1770	0.4725	−0.2528	0.150*	
C8	0.1119 (5)	0.5102 (2)	−0.1524 (6)	0.131 (3)	
H8A	0.0969	0.5390	−0.1906	0.157*	
H8B	0.0465	0.4914	−0.1604	0.157*	
C9	0.1435 (3)	0.52322 (17)	−0.0573 (5)	0.1054 (18)	
H9A	0.0858	0.5420	−0.0387	0.126*	
C10	0.2416 (3)	0.55331 (14)	−0.0445 (4)	0.0905 (14)	
H10A	0.2590	0.5649	0.0199	0.109*	
H10D	0.2290	0.5809	−0.0863	0.109*	
C11	0.3329 (3)	0.52465 (13)	−0.0658 (3)	0.0745 (12)	
H11A	0.3979	0.5444	−0.0543	0.089*	
C12	0.3515 (3)	0.48017 (12)	−0.0043 (3)	0.0668 (10)	
H12A	0.3687	0.4897	0.0615	0.080*	
H12B	0.4121	0.4624	−0.0185	0.080*	
C13	0.3074 (5)	0.50983 (18)	−0.1689 (4)	0.1148 (19)	
H13A	0.3647	0.4899	−0.1836	0.138*	
H13B	0.3004	0.5380	−0.2091	0.138*	
C14	0.1612 (3)	0.47823 (15)	0.0025 (4)	0.0888 (14)	
H14A	0.0958	0.4593	−0.0082	0.107*	
H14B	0.1776	0.4872	0.0686	0.107*	
C15	0.0612 (3)	0.30632 (12)	0.1304 (2)	0.0502 (8)	
C16	0.0685 (3)	0.25765 (13)	0.1145 (3)	0.0624 (9)	
H16A	0.1348	0.2432	0.1162	0.075*	
C17	−0.0236 (4)	0.23100 (14)	0.0963 (3)	0.0764 (11)	
H17A	−0.0204	0.1981	0.0860	0.092*	
C18	−0.1212 (3)	0.25308 (18)	0.0932 (3)	0.0794 (12)	
H18A	−0.1837	0.2349	0.0800	0.095*	
C19	−0.1273 (3)	0.30086 (17)	0.1089 (3)	0.0772 (11)	
H19A	−0.1937	0.3152	0.1071	0.093*	
C20	−0.0357 (3)	0.32805 (14)	0.1276 (3)	0.0646 (9)	
H20A	−0.0395	0.3609	0.1382	0.077*	
C21	0.0357 (3)	0.32124 (13)	−0.0982 (2)	0.0543 (8)	
C22	−0.0087 (3)	0.27858 (15)	−0.1342 (3)	0.0731 (11)	
H22A	0.0319	0.2505	−0.1279	0.088*	
C23	−0.1137 (4)	0.2781 (2)	−0.1794 (3)	0.0878 (13)	
H23A	−0.1452	0.2498	−0.2055	0.105*	
C24	−0.1705 (3)	0.3188 (2)	−0.1856 (3)	0.0879 (14)	
H24A	−0.2418	0.3192	−0.2168	0.105*	
C25	−0.1224 (3)	0.36013 (18)	−0.1453 (3)	0.0842 (13)	
H25A	−0.1634	0.3880	−0.1483	0.101*	
Cl1	0.4213 (8)	0.5932 (4)	0.1685 (5)	0.185 (4)	0.50
Cl2	0.5855 (5)	0.6034 (3)	0.3296 (5)	0.146 (3)	0.50
C26	0.5288 (10)	0.6258 (4)	0.2308 (9)	0.117 (4)	0.50
H26A	0.5822	0.6291	0.1915	0.141*	0.50
H26B	0.5038	0.6578	0.2421	0.141*	0.50

### Atomic displacement parameters (Å^2^)

**Table:**

	*U*^11^	*U*^22^	*U*^33^	*U*^12^	*U*^13^	*U*^23^
S1	0.0626 (5)	0.0619 (6)	0.0460 (5)	−0.0173 (4)	−0.0003 (4)	0.0039 (4)
S2	0.0525 (5)	0.0586 (5)	0.0574 (5)	−0.0086 (4)	0.0087 (4)	−0.0117 (4)
N1	0.0573 (18)	0.071 (2)	0.072 (2)	−0.0139 (16)	−0.0071 (15)	0.0036 (16)
O1	0.0538 (16)	0.102 (2)	0.142 (3)	0.0152 (15)	0.0085 (17)	−0.038 (2)
O2	0.0442 (13)	0.0790 (18)	0.097 (2)	−0.0072 (12)	−0.0189 (13)	0.0219 (15)
O3	0.126 (2)	0.0806 (18)	0.0551 (15)	−0.0426 (17)	0.0268 (15)	−0.0229 (14)
O4	0.0597 (15)	0.090 (2)	0.0858 (19)	−0.0064 (14)	−0.0193 (13)	0.0332 (16)
C1	0.0431 (18)	0.051 (2)	0.082 (3)	−0.0051 (16)	−0.0023 (18)	0.0075 (19)
C2	0.0397 (16)	0.0519 (18)	0.0468 (17)	−0.0052 (14)	−0.0015 (13)	−0.0003 (14)
C3	0.0434 (16)	0.0497 (18)	0.0430 (17)	−0.0056 (14)	0.0002 (13)	−0.0015 (14)
C4	0.045 (2)	0.084 (3)	0.181 (5)	0.002 (2)	−0.023 (3)	0.037 (3)
C5	0.0362 (15)	0.0476 (18)	0.059 (2)	−0.0061 (13)	0.0002 (14)	0.0027 (15)
C6	0.151 (4)	0.071 (3)	0.058 (2)	−0.037 (3)	−0.001 (3)	0.009 (2)
C7	0.230 (7)	0.073 (3)	0.059 (3)	−0.062 (4)	−0.005 (4)	0.013 (2)
C8	0.104 (4)	0.076 (3)	0.180 (6)	−0.026 (3)	−0.054 (4)	0.042 (4)
C9	0.047 (2)	0.079 (3)	0.183 (6)	0.008 (2)	0.006 (3)	0.038 (4)
C10	0.076 (3)	0.056 (2)	0.133 (4)	0.006 (2)	0.005 (3)	0.021 (2)
C11	0.049 (2)	0.055 (2)	0.114 (3)	−0.0093 (17)	0.001 (2)	0.020 (2)
C12	0.0418 (18)	0.055 (2)	0.095 (3)	−0.0078 (16)	−0.0066 (17)	0.015 (2)
C13	0.168 (5)	0.078 (3)	0.112 (4)	−0.002 (3)	0.058 (4)	0.032 (3)
C14	0.055 (2)	0.072 (3)	0.142 (4)	0.010 (2)	0.027 (2)	0.028 (3)
C15	0.0538 (19)	0.055 (2)	0.0416 (17)	−0.0116 (16)	0.0084 (14)	0.0016 (15)
C16	0.064 (2)	0.054 (2)	0.069 (2)	−0.0032 (18)	0.0145 (18)	0.0095 (18)
C17	0.096 (3)	0.057 (2)	0.079 (3)	−0.023 (2)	0.026 (2)	−0.002 (2)
C18	0.069 (3)	0.099 (3)	0.073 (3)	−0.034 (2)	0.022 (2)	−0.002 (2)
C19	0.059 (2)	0.096 (3)	0.081 (3)	−0.007 (2)	0.024 (2)	−0.005 (2)
C20	0.067 (2)	0.063 (2)	0.067 (2)	−0.0033 (19)	0.0208 (18)	−0.0039 (18)
C21	0.0552 (19)	0.068 (2)	0.0389 (17)	−0.0183 (18)	0.0073 (14)	−0.0029 (16)
C22	0.074 (2)	0.075 (3)	0.071 (2)	−0.027 (2)	0.014 (2)	−0.020 (2)
C23	0.079 (3)	0.102 (4)	0.080 (3)	−0.039 (3)	0.010 (2)	−0.025 (3)
C24	0.063 (3)	0.135 (4)	0.060 (2)	−0.040 (3)	−0.0044 (19)	0.001 (3)
C25	0.058 (2)	0.100 (3)	0.086 (3)	−0.011 (2)	−0.008 (2)	0.011 (3)
Cl1	0.187 (7)	0.233 (7)	0.133 (5)	−0.043 (5)	0.022 (4)	−0.097 (5)
Cl2	0.101 (3)	0.159 (4)	0.154 (5)	0.051 (3)	−0.035 (3)	−0.050 (4)
C26	0.111 (11)	0.150 (9)	0.096 (9)	0.013 (7)	0.029 (6)	0.009 (7)

### Geometric parameters (Å, °)

**Table:**

S1—O4	1.426 (3)	C10—H10D	0.9700
S1—O3	1.427 (3)	C11—C12	1.515 (5)
S1—C15	1.759 (3)	C11—C13	1.518 (7)
S1—C3	1.799 (3)	C11—H11A	0.9800
S2—C21	1.764 (3)	C12—H12A	0.9700
S2—C3	1.800 (3)	C12—H12B	0.9700
N1—C21	1.327 (5)	C13—H13A	0.9700
N1—C25	1.338 (5)	C13—H13B	0.9700
O1—C1	1.187 (5)	C14—H14A	0.9700
O2—C1	1.326 (4)	C14—H14B	0.9700
O2—C4	1.440 (5)	C15—C20	1.366 (5)
C1—C2	1.505 (5)	C15—C16	1.379 (5)
C2—C3	1.537 (4)	C16—C17	1.367 (5)
C2—C5	1.552 (4)	C16—H16A	0.9300
C2—H2A	0.9800	C17—C18	1.376 (6)
C3—H3A	0.9800	C17—H17A	0.9300
C4—H4A	0.9600	C18—C19	1.353 (6)
C4—H4B	0.9600	C18—H18A	0.9300
C4—H4C	0.9600	C19—C20	1.370 (5)
C5—C6	1.508 (5)	C19—H19A	0.9300
C5—C14	1.524 (5)	C20—H20A	0.9300
C5—C12	1.526 (4)	C21—C22	1.372 (5)
C6—C7	1.579 (6)	C22—C23	1.366 (6)
C6—H6A	0.9700	C22—H22A	0.9300
C6—H6B	0.9700	C23—C24	1.337 (6)
C7—C8	1.530 (9)	C23—H23A	0.9300
C7—C13	1.570 (8)	C24—C25	1.376 (6)
C7—H7A	0.9800	C24—H24A	0.9300
C8—C9	1.401 (9)	C25—H25A	0.9300
C8—H8A	0.9700	Cl1—C26^i^	1.726 (14)
C8—H8B	0.9700	Cl1—C26	1.737 (15)
C9—C10	1.483 (6)	Cl2—C26	1.594 (14)
C9—C14	1.513 (6)	Cl2—C26^i^	1.663 (15)
C9—H9A	0.9800	C26—H26A	0.9700
C10—C11	1.488 (6)	C26—H26B	0.9700
C10—H10A	0.9700		
			
O4—S1—O3	118.38 (18)	H10A—C10—H10D	108.2
O4—S1—C15	109.21 (16)	C10—C11—C12	110.8 (4)
O3—S1—C15	108.75 (17)	C10—C11—C13	108.8 (4)
O4—S1—C3	108.91 (16)	C12—C11—C13	109.4 (4)
O3—S1—C3	106.81 (16)	C10—C11—H11A	109.2
C15—S1—C3	103.79 (14)	C12—C11—H11A	109.2
C21—S2—C3	100.30 (16)	C13—C11—H11A	109.2
C21—N1—C25	116.5 (3)	C11—C12—C5	111.2 (3)
C1—O2—C4	115.7 (4)	C11—C12—H12A	109.4
O1—C1—O2	123.8 (3)	C5—C12—H12A	109.4
O1—C1—C2	124.9 (3)	C11—C12—H12B	109.4
O2—C1—C2	111.3 (3)	C5—C12—H12B	109.4
C1—C2—C3	111.2 (3)	H12A—C12—H12B	108.0
C1—C2—C5	113.2 (3)	C11—C13—C7	107.8 (4)
C3—C2—C5	114.4 (2)	C11—C13—H13A	110.1
C1—C2—H2A	105.7	C7—C13—H13A	110.1
C3—C2—H2A	105.7	C11—C13—H13B	110.1
C5—C2—H2A	105.7	C7—C13—H13B	110.1
C2—C3—S1	108.4 (2)	H13A—C13—H13B	108.5
C2—C3—S2	117.5 (2)	C9—C14—C5	111.6 (4)
S1—C3—S2	109.63 (17)	C9—C14—H14A	109.3
C2—C3—H3A	106.9	C5—C14—H14A	109.3
S1—C3—H3A	106.9	C9—C14—H14B	109.3
S2—C3—H3A	106.9	C5—C14—H14B	109.3
O2—C4—H4A	109.5	H14A—C14—H14B	108.0
O2—C4—H4B	109.5	C20—C15—C16	121.2 (3)
H4A—C4—H4B	109.5	C20—C15—S1	118.9 (3)
O2—C4—H4C	109.5	C16—C15—S1	119.9 (3)
H4A—C4—H4C	109.5	C17—C16—C15	118.8 (4)
H4B—C4—H4C	109.5	C17—C16—H16A	120.6
C6—C5—C14	108.0 (3)	C15—C16—H16A	120.6
C6—C5—C12	109.3 (3)	C16—C17—C18	119.7 (4)
C14—C5—C12	106.3 (3)	C16—C17—H17A	120.1
C6—C5—C2	114.5 (3)	C18—C17—H17A	120.1
C14—C5—C2	109.0 (3)	C19—C18—C17	120.9 (4)
C12—C5—C2	109.5 (2)	C19—C18—H18A	119.5
C5—C6—C7	109.9 (3)	C17—C18—H18A	119.5
C5—C6—H6A	109.7	C18—C19—C20	120.0 (4)
C7—C6—H6A	109.7	C18—C19—H19A	120.0
C5—C6—H6B	109.7	C20—C19—H19A	120.0
C7—C6—H6B	109.7	C15—C20—C19	119.2 (4)
H6A—C6—H6B	108.2	C15—C20—H20A	120.4
C8—C7—C13	110.1 (4)	C19—C20—H20A	120.4
C8—C7—C6	109.5 (5)	N1—C21—C22	123.2 (3)
C13—C7—C6	105.0 (5)	N1—C21—S2	118.9 (2)
C8—C7—H7A	110.7	C22—C21—S2	117.7 (3)
C13—C7—H7A	110.7	C23—C22—C21	118.8 (4)
C6—C7—H7A	110.7	C23—C22—H22A	120.6
C9—C8—C7	111.2 (4)	C21—C22—H22A	120.6
C9—C8—H8A	109.4	C24—C23—C22	119.2 (4)
C7—C8—H8A	109.4	C24—C23—H23A	120.4
C9—C8—H8B	109.4	C22—C23—H23A	120.4
C7—C8—H8B	109.4	C23—C24—C25	119.4 (4)
H8A—C8—H8B	108.0	C23—C24—H24A	120.3
C8—C9—C10	109.9 (5)	C25—C24—H24A	120.3
C8—C9—C14	109.2 (5)	N1—C25—C24	122.9 (5)
C10—C9—C14	111.6 (4)	N1—C25—H25A	118.6
C8—C9—H9A	108.7	C24—C25—H25A	118.6
C10—C9—H9A	108.7	Cl2—C26—Cl1	115.6 (7)
C14—C9—H9A	108.7	Cl2—C26—H26A	108.4
C9—C10—C11	110.0 (4)	Cl1—C26—H26A	108.4
C9—C10—H10A	109.7	Cl2—C26—H26B	108.4
C11—C10—H10A	109.7	Cl1—C26—H26B	108.4
C9—C10—H10D	109.7	H26A—C26—H26B	107.4
C11—C10—H10D	109.7		
			
C4—O2—C1—O1	−5.1 (5)	C14—C5—C12—C11	−58.7 (4)
C4—O2—C1—C2	176.1 (3)	C2—C5—C12—C11	−176.3 (3)
O1—C1—C2—C3	57.9 (5)	C10—C11—C13—C7	−57.4 (5)
O2—C1—C2—C3	−123.3 (3)	C12—C11—C13—C7	63.9 (5)
O1—C1—C2—C5	−72.6 (5)	C8—C7—C13—C11	53.4 (6)
O2—C1—C2—C5	106.2 (3)	C6—C7—C13—C11	−64.3 (5)
C1—C2—C3—S1	80.7 (3)	C8—C9—C14—C5	63.4 (5)
C5—C2—C3—S1	−149.4 (2)	C10—C9—C14—C5	−58.3 (6)
C1—C2—C3—S2	−44.2 (3)	C6—C5—C14—C9	−59.5 (5)
C5—C2—C3—S2	85.6 (3)	C12—C5—C14—C9	57.7 (5)
O4—S1—C3—C2	−69.3 (2)	C2—C5—C14—C9	175.6 (4)
O3—S1—C3—C2	59.7 (3)	O4—S1—C15—C20	164.6 (3)
C15—S1—C3—C2	174.5 (2)	O3—S1—C15—C20	34.1 (3)
O4—S1—C3—S2	60.2 (2)	C3—S1—C15—C20	−79.3 (3)
O3—S1—C3—S2	−170.85 (17)	O4—S1—C15—C16	−17.7 (3)
C15—S1—C3—S2	−56.0 (2)	O3—S1—C15—C16	−148.2 (3)
C21—S2—C3—C2	−138.9 (2)	C3—S1—C15—C16	98.3 (3)
C21—S2—C3—S1	96.70 (18)	C20—C15—C16—C17	−0.4 (5)
C1—C2—C5—C6	73.5 (4)	S1—C15—C16—C17	−178.0 (3)
C3—C2—C5—C6	−55.4 (4)	C15—C16—C17—C18	0.7 (6)
C1—C2—C5—C14	−165.5 (3)	C16—C17—C18—C19	−0.7 (6)
C3—C2—C5—C14	65.6 (4)	C17—C18—C19—C20	0.5 (6)
C1—C2—C5—C12	−49.7 (4)	C16—C15—C20—C19	0.2 (5)
C3—C2—C5—C12	−178.5 (3)	S1—C15—C20—C19	177.9 (3)
C14—C5—C6—C7	55.1 (5)	C18—C19—C20—C15	−0.3 (6)
C12—C5—C6—C7	−60.2 (5)	C25—N1—C21—C22	−1.3 (5)
C2—C5—C6—C7	176.6 (4)	C25—N1—C21—S2	175.3 (3)
C5—C6—C7—C8	−54.8 (6)	C3—S2—C21—N1	32.5 (3)
C5—C6—C7—C13	63.5 (6)	C3—S2—C21—C22	−150.6 (3)
C13—C7—C8—C9	−56.3 (6)	N1—C21—C22—C23	2.5 (6)
C6—C7—C8—C9	58.7 (5)	S2—C21—C22—C23	−174.2 (3)
C7—C8—C9—C10	60.8 (5)	C21—C22—C23—C24	−1.3 (6)
C7—C8—C9—C14	−61.9 (5)	C22—C23—C24—C25	−0.8 (7)
C8—C9—C10—C11	−65.1 (5)	C21—N1—C25—C24	−1.0 (6)
C14—C9—C10—C11	56.2 (6)	C23—C24—C25—N1	2.1 (7)
C9—C10—C11—C12	−57.0 (5)	Cl1^i^—Cl2—C26—Cl1	13 (4)
C9—C10—C11—C13	63.4 (5)	C26^i^—Cl2—C26—Cl1	−62.4 (9)
C10—C11—C12—C5	60.2 (5)	Cl2^i^—Cl1—C26—Cl2	127 (3)
C13—C11—C12—C5	−59.8 (5)	C26^i^—Cl1—C26—Cl2	64.3 (11)
C6—C5—C12—C11	57.6 (4)		

Symmetry codes: (i) −*x*+1, *y*, −*z*+1/2.
